# The Prognostic Value of Dynamic Changes in Prognostic Nutritional Index (PNI) During Treatment in Lung Cancer: Is Improvement a Better Predictor than Baseline?

**DOI:** 10.3390/nu18040644

**Published:** 2026-02-16

**Authors:** Eren Mingsar, İlhan Öztop, Sinan Ünal

**Affiliations:** Department of Medical Oncology, Faculty of Medicine, Dokuz Eylul University, İnciraltı, İzmir 35340, Türkiye; ilhan.oztop@deu.edu.tr (İ.Ö.); sinan.unal@deu.edu.tr (S.Ü.)

**Keywords:** lung cancer, prognostic nutritional index, dynamic biomarker, survival, nutritional status, immunonutrition

## Abstract

Objective: Although the baseline prognostic nutritional index (PNI) is a well-known prognostic factor in lung cancer, the clinical significance of its fluctuation during treatment remains unclear. This study aimed to evaluate the prognostic value of dynamic changes in the PNI and to determine whether improvements in nutritional and immune status are correlated with survival outcomes. Methods: A total of 478 patients diagnosed with lung cancer were retrospectively analyzed. The PNI was calculated on the basis of serum albumin levels and total lymphocyte counts. The baseline value was termed PNI1, and the posttreatment value was termed PNI2. The dynamic changes in PNIΔ were categorized as increased, stable, or decreased. Relationships between these dynamic parameters and Overall Survival and Progression-Free Survival were assessed using Kaplan–Meier and Cox regression analyses. Results: The median follow-up was 19.9 months. Patients with higher PNI1 and PNI2 scores had significantly longer OS and PFS. Notably, patients who demonstrated an increase in PNIΔ during the treatment course had significantly longer overall survival than those with stable or decreased scores (*p* = 0.023). Multivariate analysis revealed that while cancer type and the posttreatment PNI (PNI2) were identified as independent prognostic factors (*p* = 0.007 for PNI2), the dynamic improvement in the PNI emerged as a critical indicator of a better clinical trajectory according to univariate analysis. Conclusion: This study demonstrates that the PNI is not merely a static baseline marker but also a dynamic biomarker that reflects the host’s response to treatment and disease. An increase in PNI values during treatment is associated with improved survival, suggesting that dynamic monitoring of nutritional and immune status provides valuable prognostic information for patient management in lung cancer patients.

## 1. Introduction

Lung cancer remains among the most commonly diagnosed cancers worldwide and continues to be a leading cause of cancer-related mortality. According to data from 2020, more than 2.2 million new cases of lung cancer were reported globally, with approximately 1.8 million deaths attributed to the disease. The overall prognosis of lung cancer is poor, with five-year survival rates generally ranging between 10% and 20%. Most patients are diagnosed at an advanced stage, which significantly limits treatment success and survival outcomes [1]. In Turkey, lung cancer remains a significant public health burden. Recent national data indicate that it is the leading cause of cancer-related mortality and incidence, particularly among men, highlighting the urgent need for effective prognostic markers in this population [2]. Although recent advances in targeted therapies and immunotherapy have led to improvements in survival, reliably predicting prognosis in lung cancer patients remains a critical need. In the diagnostic and treatment process, in addition to clinicopathological factors such as age, sex, performance status, weight loss, and tumor stage, laboratory-based biomarkers play important roles in predicting prognosis [3,4,5,6].

Common conditions in cancer patients, such as anorexia, weight loss, and cachexia, not only deteriorate quality of life but also negatively affect survival. These findings emphasize the critical role of nutritional status in influencing patient outcomes [7,8].

Although complex systems for evaluating nutritional status that incorporate clinical, laboratory, and anthropometric parameters exist, simpler and more practical scoring systems have been developed [9,10]. One such system is the prognostic nutritional index (PNI), which is calculated by combining serum albumin levels and total lymphocyte count and serves as a practical tool reflecting a patient’s nutritional and immune status. Initially developed to assess surgical risk, the PNI score has since been used as a prognostic indicator in various malignancies. However, data concerning the impact of dynamic changes in the PNI during treatment on prognosis remain limited [11].

This study aimed to investigate the effects of pre- and posttreatment PNI values on treatment response and survival in patients diagnosed with lung cancer and to evaluate the prognostic significance of dynamic PNI monitoring.

## 2. Materials and Methods

### 2.1. Study Population and Baseline Data

This retrospective cohort study included patients diagnosed with lung cancer who were followed and treated at the Department of Medical Oncology, Dokuz Eylul University Faculty of Medicine, between January 2012 and December 2017.

The following criteria were applied for patient selection: histopathological confirmation of lung cancer at the time of diagnosis; availability of adequate laboratory data before and after treatment; and the absence of systemic conditions that could affect the treatment process, such as severe infections, hematological malignancies, or uncontrolled autoimmune diseases.

Patient demographic data (age, sex, and smoking status), histological type and stage of the tumor, surgical and systemic treatment details, laboratory parameters (serum albumin concentration and total lymphocyte count), treatment response, progression, and date of death were obtained from hospital records. No specific time cutoff was applied for exclusion on the basis of follow-up duration. The follow-up period continued until the patient’s death or the last recorded clinical visit.

### 2.2. PNI Analyses

The prognostic nutritional index (PNI) was calculated using the standard formula established by Onodera et al. [12]: PNI = 10 × serum albumin (g/dL) + 0.005 × total lymphocyte count (per mm^3^) [12].

The baseline PNI, termed PNI1, was determined on the basis of laboratory results obtained within one week prior to surgery or before the initiation of first-line systemic therapy. The posttreatment PNI, termed PNI2, was assessed using data obtained after the second postoperative week for surgical patients or two weeks following the completion of first-line chemotherapy for patients receiving systemic treatment. The dynamic change between these time points was termed PNIΔ (PNI2-PNI1).

Receiver operating characteristic (ROC) curve analysis was conducted to evaluate the prognostic discriminatory ability of the PNI. The analysis yielded an area under the curve (AUC) of 0.648 (95% CI: 0.598–0.699; *p* < 0.001), with an optimal mathematical cutoff value of 49.3 determined by the Youden index (0.247). Although this optimal threshold was higher than the cutoff of 40 originally proposed by Onodera et al., relying on a single dataset-specific value carries a risk of statistical overfitting. Therefore, to ensure consistency with established risk stratification models and to evaluate the graded prognostic impact of nutritional status, patients were stratified into three standard categories on the basis of PNI score: <45, 45–50, and >50.

### 2.3. Response Evaluation

Treatment responses of all patients were evaluated according to the Response Evaluation Criteria in Solid Tumors (RECIST) 1.1 protocol. Response categories included complete response, partial response, stable disease, and progressive disease. The tumor response was evaluated according to the Response Evaluation Criteria in Solid Tumors (RECIST) version 1.1 criteria using contrast-enhanced computed tomography (CT) or positron emission tomography-computed tomography (PET-CT) scans [13]. Response categories included complete response, partial response, stable disease, and progressive disease.

### 2.4. Statistical and Survival Analyses

Statistical analyses were performed using SPSS version 26.0 (IBM Corp., Armonk, NY, USA). Continuous variables are expressed as the mean ± standard deviation and median (25th–75th percentiles), and categorical variables are expressed as the frequency and percentage. The distribution of the data was assessed with the Kolmogorov–Smirnov test. The relationships between clinicopathological variables and PNI groups were analyzed using one-way ANOVA.

Survival analyses were conducted using the Kaplan–Meier method, and differences between groups were compared with the log-rank test. Overall survival (OS), disease-free survival (DFS), and progression-free survival (PFS) were defined. To identify independent prognostic factors affecting survival, multivariate analysis was performed using the Cox proportional hazards regression model (forward-conditional method). A *p* value of <0.05 was considered to indicate statistical significance. The optimal cutoff values for the PNI were determined using receiver operating characteristic (ROC) curve analysis. The Youden index (sensitivity + specificity − 1) was used to identify the threshold with the maximum predictive power for overall survival.

## 3. Results

### 3.1. Demographic and Clinical Results

A total of 478 lung cancer patients were included in the study. Among them, 382 (80%) were male and 96 (20%) were female. The mean age was 63.7 ± 10.1 years, and the median age was 64 years. Among the patients, 83.6% were diagnosed with non-small cell lung cancer (NSCLC), while 16.4% had small cell lung cancer (SCLC).

Among the NSCLC patients, the most common histological subtype was adenocarcinoma (42%), followed by squamous cell carcinoma (30%). At diagnosis, 26% of the NSCLC patients had stage I–II disease, 18% had stage III disease, and 56% had stage IV disease. In contrast, 28% of SCLC patients were at the limited stage, and 72% were at the extensive stage. The median follow-up duration for the entire cohort was 19.9 months, and the median overall survival was 24.3 months. The demographic and clinical characteristics of the patients are summarized in Table 1.

### 3.2. PNI Results

The pretreatment PNI1 values measured across all patients ranged from a minimum of 26.5 to a maximum of 111.0, with a median of 47.7. The median PNI1 value was 48.5 in patients with non-small cell lung cancer (NSCLC) and 47.9 in those with small cell lung cancer (SCLC).

Posttreatment PNI2 values ranged from a minimum of 18.5 to a maximum of 64.6, with a median value of 45.3 for the entire cohort. The median PNI2 values were 45.0 in NSCLC patients and 45.7 in SCLC patients. The difference between PNI1 and PNI2, referred to as PNIΔ, had a median value of 3.9 across all patients. On the basis of PNIΔ, patients were grouped as follows: 16% (*n* = 54) showed increased PNIΔ, 43% (*n* = 152) showed stable PNIΔ, and 41% (*n* = 145) showed decreased PNIΔ. All the PNI values and their distributions are presented in Table 2.

### 3.3. Response and Survival Outcomes

The treatment response of 297 NSCLC patients and 78 SCLC patients who received systemic therapy was evaluated according to the RECIST 1.1 criteria. Among the NSCLC patients, the overall response rate (ORR) to first-line chemotherapy was 64.6%, with a complete response (CR) rate of 2.5% and a partial response (PR) rate of 62.1%. In SCLC patients, the ORR was 39.8%, with 10.3% achieving CR and 29.5% achieving PR.

**Figure 1:**
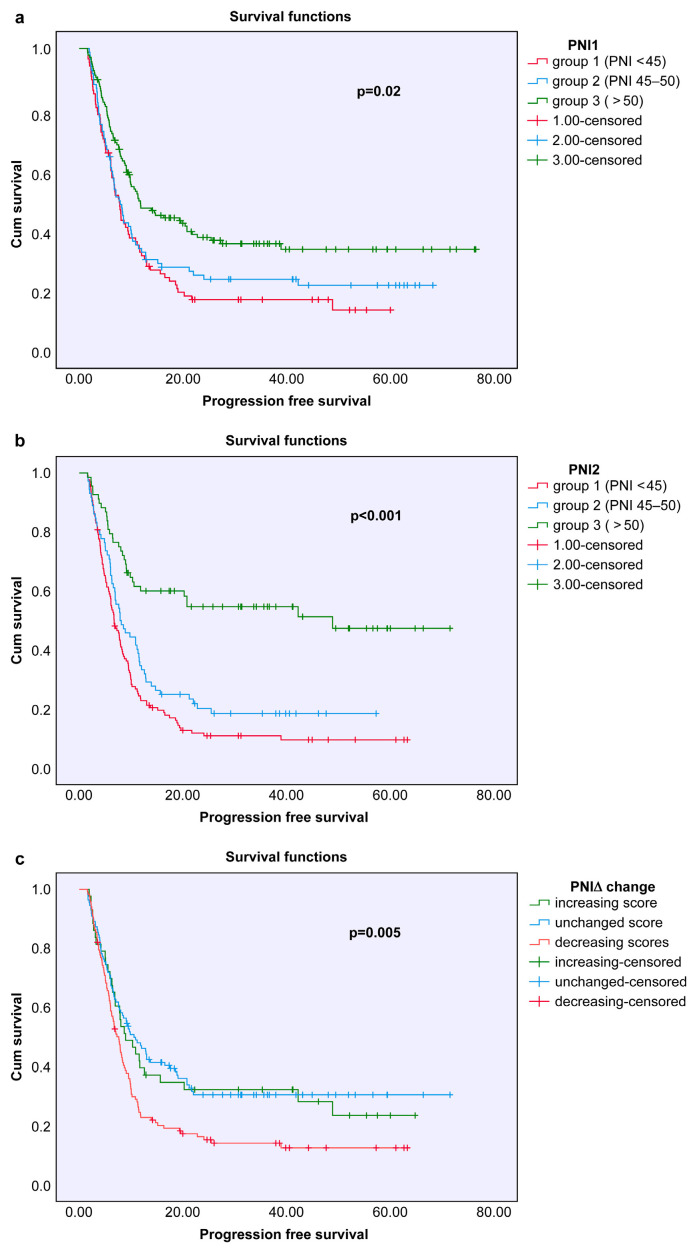
Kaplan–Meier analysis of progression-free survival (PFS) based on the prognostic nutritional index (PNI). Subfigure (**a**) shows the PFS distribution stratified by baseline PNI score: Group 1 (PNI < 45), Group 2 (PNI 45–50), and Group 3 (PNI > 50). Subfigure (**b**) presents the PFS distribution according to follow-up PNI categories using the same cutoff values. The differences in PFS based on the change in PNI (ΔPNI) during follow-up are shown in Subfigure (**c**) and can be divided into three groups: increasing score, unchanged score, and decreasing score. Significant differences in progression-free survival were observed among the groups in all analyses (*p* < 0.05).

For the 103 NSCLC patients who underwent surgery at an early stage, the median disease-free survival (DFS) was 30.6 months, and the median overall survival (OS) was 36.3 months. In patients with locally advanced or metastatic NSCLC, the median progression-free survival (PFS) was 7.0 months, and the median OS was 16.6 months. In SCLC patients, the median PFS was 7.8 months, while the median OS was 14.1 months. Progression-free survival analyses according to age, sex, and cancer subtype subgroup are presented in Figure 1.

### 3.4. Relationships Between PNI Values and Survival

In survival analyses based on the PNI1, PNI2, and PNIΔ groups, no statistically significant relationship was observed between disease-free survival (DFS) and PNI scores in early-stage NSCLC patients (PNI1: *p* = 0.53; PNI2: *p* = 0.54; PNIΔ: *p* = 0.23). However, in patients with locally advanced or metastatic NSCLC, a significant association was found between PNI1 and PNI2 values and progression-free survival (PFS) (PNI1: *p* = 0.027; PNI2: *p* = 0.006), whereas PNIΔ was not significantly related (*p* = 0.42). Among SCLC patients, no significant association was identified between PNI score and PFS (PNI1: *p* = 0.27; PNI2: *p* = 0.54; PNIΔ: *p* = 0.87).

**Figure 2:**
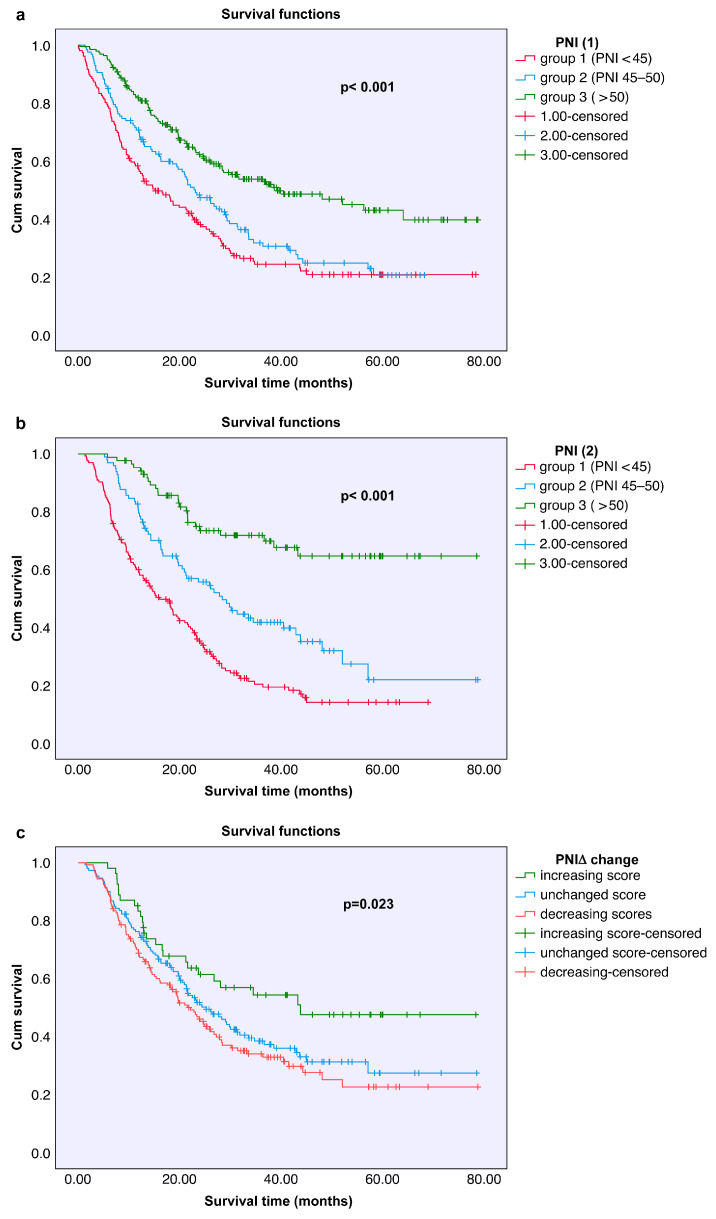
This figure illustrates the results of the Kaplan–Meier survival analysis based on the prognostic nutritional index (PNI). Subfigure (**a**) shows the survival distribution categorized by baseline PNI values: Group 1 (PNI < 45), Group 2 (PNI between 45 and 50), and Group 3 (PNI > 50). Subfigure (**b**) presents the survival distribution according to the PNI values measured during follow-up using the same group classifications. Subfigure (**c**) compares the survival outcomes according to the change in PNI (ΔPNI), which can be divided into three groups: increasing score, unchanged score, and decreasing score. Statistically significant differences in survival were observed among the groups in each analysis (*p* < 0.05).

Overall survival analyses revealed that patients with higher PNI1 and PNI2 scores had significantly longer survival durations (both *p* < 0.001). Additionally, patients whose PNIΔ increased after treatment had significantly longer survival than those whose PNIΔ was stable or decreased (*p* = 0.023). Survival analyses according to PNI groups are presented in Table 3 and Table 4, and Kaplan–Meier curves are shown in Figure 2.

Univariate analyses revealed significant associations between overall survival and PNI1, PNI2, PNIΔ, tumor type, patient sex, and patient age. On the basis of these findings, a multivariate analysis covering the entire patient group was conducted using the Cox regression model. According to the results of the multivariate analysis, two independent variables significantly affected survival duration: cancer type and posttreatment PNI2 score. Patients with non-small cell lung cancer (NSCLC) histology had significantly longer survival (*p* = 0.003). Furthermore, patients with a PNI2 score <45 had notably shorter survival (*p* = 0.007). These results indicate that both the histopathological type of cancer and nutritional status independently influence prognosis. The detailed results of the multivariate Cox regression analysis are presented in Table 5.

## 4. Discussion

In this study, we investigated the relationships between the prognostic nutritional index (PNI) values calculated before and after treatment and overall survival and treatment response in patients diagnosed with lung cancer. Our findings revealed that high PNI1 and PNI2 values were positively associated with overall survival. Additionally, patients whose PNIΔ scores increased had significantly longer survival durations. These results support the prognostic significance of nutritional and immune status. The PNI was originally proposed by Onodera et al. for the prediction of postoperative complications in patients undergoing gastrointestinal surgery [12]. With respect to the timing of the posttreatment assessment, PNI2 was measured two weeks after treatment completion. This time point was chosen to evaluate the early nutritional and immunological recovery status of the patients before the initiation of subsequent therapeutic lines or long-term convalescence. However, we acknowledge that acute treatment-induced inflammation might persist at this stage, potentially influencing albumin levels and lymphocyte counts. While a later measurement (e.g., at 3 or 6 months) could reflect a more stabilized baseline, the two-week mark offers clinical utility as an early indicator for identifying high-risk patients who may require immediate nutritional intervention.

Subsequent studies have demonstrated the prognostic value of the PNI in various cancer types, especially gastrointestinal, pancreatic, hepatocellular, and hematologic malignancies [14,15,16]. In a study conducted by K. Migita et al. on patients with gastric cancer, a PNI cutoff value of 48 was identified via receiver operating characteristic (ROC) analysis [17]. Clinical studies have shown that PNI scores can be stratified on the basis of patient population. Similarly, in our study, dynamic calculation of the PNI was potentially effective for evaluating prognosis both at the individual level and in general populations. An examination of the association between pretreatment PNI1 values and overall survival revealed that patients with higher PNI1 scores had significantly longer survival. The log-rank test revealed a statistically significant difference (χ^2^ = 30.7; *p* = 0.001). Although studies encompassing all lung cancer patients remain limited in the literature, the number of such investigations is gradually increasing. For example, a study by Oku et al. (2023) also revealed that the pretreatment PNI is a strong predictor of survival [18].

High posttreatment PNI2 scores were also associated with long overall survival in our cohort. Moreover, multivariate analysis revealed that PNI2 is an independent prognostic factor. This finding is supported by the study conducted by Nishihara-Kato et al. [19]. Upon comparing the PNI values before and after treatment, 353 patients were categorized into groups on the basis of increasing, decreasing, and stable PNIΔ scores. Kaplan–Meier analysis indicated that patients with increased PNIΔ scores had significantly longer overall survival (*p* = 0.023). Although dynamic changes in the PNI (PNIΔ) were significantly associated with overall survival according to the univariate analysis, it did not emerge as an independent prognostic factor in the multivariate analysis. Therefore, PNIΔ should be interpreted as a complementary and exploratory biomarker rather than a definitive independent predictor. Similarly, Lei et al. (2024) reported that changes in the PNI were associated with progression-free survival [20].

A study involving NSCLC patients treated with PD-1 inhibitors revealed that the PNI is a predictor of early progression [21]. Similarly, a meta-analysis evaluating NSCLC patients receiving immune checkpoint-based therapy demonstrated a significant association between pretreatment PNI and progression-free survival [22].

Collectively, these findings highlight that while the posttreatment PNI serves as a robust independent prognostic marker, evaluating dynamic changes (PNIΔ) provides valuable additional insight into the patient’s clinical trajectory. Thus, the PNI serves as an independent prognostic marker for predicting overall survival, progression-free survival, and treatment response. Our findings are in accordance with the current literature.

## 5. Conclusions and Recommendations

In this study, the relationships between prognostic nutritional index (PNI) values, which were calculated before and after treatment, and survival and treatment response in patients with lung cancer were comprehensively assessed. Our findings indicate that high PNI1 and PNI2 values are positively associated with overall survival and progression-free survival. Furthermore, increases in the PNI during the treatment course may indicate a better prognosis, which aligns with findings from the contemporary literature. Nonetheless, the study has several limitations. First, its retrospective and single-center design may affect the generalizability of the results. Second, the exclusion of patients with early mortality introduces potential immortal time bias, as those who died before the second assessment could not be included in the dynamic analysis. Third, the study population was heterogeneous and included both NSCLC and SCLC patients with varying disease stages. Finally, although the PNI is a nutritional marker, we could not perform a detailed adjustment for potential confounders such as body mass index (BMI) and comorbidities such as diabetes mellitus, which are known to influence systemic inflammation and nutritional status. These factors should be considered in future prospective studies.

In conclusion, the PNI may serve as a valuable and dynamic biomarker for predicting prognosis and treatment response in patients with lung cancer, regardless of cancer subtype. The findings from this study suggest that monitoring the PNI at baseline and during treatment may provide meaningful insights for patient management. In particular, pre- and posttreatment changes in the PNI could help estimate treatment response and disease trajectory. Future research should focus on larger, multicenter prospective studies to further explore the role of the PNI in treatment planning and individualized oncology approaches. Furthermore, combining the PNI with other biomarkers may increase its predictive power.

## Figures and Tables

**Table 1:** Lung carcinoma patient demographic data at the time of diagnosis.

	N = 478	%	Mean	Median	Std.	Minimum	Maximum
Gender (Female/Male)	96/382	20/80					
Smoking	360/118	75/25					
Lung cancer type Small cell/Non-small cell	78/400	17/83					
Surgically operable/Inoperable	139/339	29/71					
Age			63.7	64.0	10.1	20.0	100.0
Body mass index			40.0	39.2	7.4	16.0	68.0
Hemoglobin (gr/dL)			12.5	13.0	2.0	5.7	17.6
Albumin (gr/dL)			3.7	3.8	0.4	2.2	4.9
Lymphocyte (10^3^/μL)			1.95	1.8	1.01	0.2	15.0
Leukocyte (10^3^/μL)			9.5	9.2	3.4	1.0	43.1
PNI1	478		47.7	48.0	7.5	26.5	111.0
PNI2	351		44.2	45.3	8.3	18.5	64.6
PNIΔ (increased/unchanged/decreased)	54/152/145						

PNI1: initial measurement of the prognostic nutritional index, PNI2: second measurement of the prognostic nutritional index, PNIΔ: change in the prognostic nutritional index (increased/unchanged/decreased).

**Table 2:** PNI scores of all patients.

	Highest	Lowest	Median
PNI1 of all patients	111.0	26.5	47.7
PNI1 of NSCLC patients	74.0	26.5	48.5
PNI1 of SCLC patients	111.0	32.5	47.9
PNI2 of all patients	64.6	18.5	45.3
PNI2 of NSCLC patients	63.5	18.5	45.0
PNI2 of SCLC patients	64.6	19.1	45.7
PNIΔ of all patients	80.0	−18.0	3.9
PNIΔ of NSCLC patients	33.5	−18.0	4.0
PNIΔ of SCLC patients	80.5	−14.5	4.1

PNI1: initial measurement of the prognostic nutritional index, PNI2: second measurement of the prognostic nutritional index, PNIΔ: change in the prognostic nutritional index (increased/unchanged/decreased).

**Table 3:** Relationships between PNI and progression-free survival in NSCLC patients.

	Mean ± (SD) Months	Median Months	*p* (Log-Rank)
PNI1			
Group 1 (<45)	10.9 ± 1.5	6.6	
Group 2 (45–50)	10.6 ± 1.8	6.4	0.027
Group 3 (>50)	14.5 ± 3.1	7.9	
PNI2			
Group 1 (<45)	9.4 ± 1.1	6.1	
Group 2 (45–50)	14.9 ± 2.3	7.6	0.006
Group 3 (>50)	20.4 ± 4.6	8.9	
PNIΔ (score change)			
decreased scores	16.1 ±.3.6	6.9	
unchanged score	11.9 ± 1.7	6.8	0.42
increased score	11.1 ± 1.5	6.5	

PNI1: initial measurement of the prognostic nutritional index, PNI2: second measurement of the prognostic nutritional index, PNIΔ: change in the prognostic nutritional index (increased/unchanged/decreased).

**Table 4:** Factors affecting survival in lung cancer patients.

	Mean ± (SD) Months	Median Months	*p* (Log-Rank)
Small cell	25 ± 2.9	16.7	<0.001
Non-small cell	37.9 ± 1.68	27.2	
Age ≤64	39.38 ± 2.18	28.6	0.017
Age >64	32 ± 2.01	21.3	
Male	33.7 ± 1.6	22.5	0.001
Female	45.4 ± 3.4	43.7	
PNI1			
Group 1 (<45)	27.5 ± 2.3	15.1	
Group 2 (45–50)	29.9 ± 2.2	23	<0.001
Group 3 (>50)	45 ± 2.4	39.2	
PNI2			
Group 1 (<45)	23.5 ± 1.7	16.1	
Group 2 (45–50)	36.9 ± 3.1	28.3	<0.001
Group 3 (>50)	58.7 ± 3.3	50	
PNIΔ score change			
PNIΔ decreased	32.7 ± 2.6	21.5	
PNIΔ unchanged	36.3 ± 2.6	25.2	0.023
PNIΔ increased	47.2 ± 4.4	43.7	

PNI1: initial measurement of the prognostic nutritional index, PNI2: second measurement of the prognostic nutritional index, PNIΔ: change in the prognostic nutritional index (increased/unchanged/decreased).

**Table 5:** Multivariate Cox regression analysis of prognostic factors affecting overall survival in patients with lung cancer. Both posttreatment PNI (PNI2) and histological cancer type (NSCLC vs. SCLC) were found to be independent predictors of survival.

	B	SE	Wald	df	Sig.	Exp(B)
Lung cancer type	−0.517	0.175	8.757	1	0.003	0.597
AGE	0.072	0.143	0.254	1	0.614	1.075
Gender	−0.332	0.203	2.672	1	0.102	0.718
PNI1 Group 1			2.674	2	0.263	
PNI1 Group 2	0.200	0.466	0.183	1	0.668	1.221
PNI1 Group 3	0.322	0.232	1.930	1	0.165	1.380
PNI2 Group 1			9.925	2	0.007	
PNI2 Group 2	1.514	0.483	9.833	1	0.002	4.546
PNI2 Group 3	0.938	0.330	8.081	1	0.004	2.555
PNIΔ increased			0.858	2	0.651	
PNIΔ stable	0.101	0.622	0.026	1	0.871	1.106
PNIΔ decreased	0.199	0.351	0.323	1	0.570	1.221

PNI1: initial measurement of the prognostic nutritional index, PNI2: second measurement of the prognostic nutritional index, PNIΔ: change in the prognostic nutritional index (increased/unchanged/decreased).

## Data Availability

The data presented in this study are available on request from the corresponding author due to privacy and ethical restrictions.
